# Venom Atypical Extracellular Vesicles as Interspecies Vehicles of Virulence Factors Involved in Host Specificity: The Case of a *Drosophila* Parasitoid Wasp

**DOI:** 10.3389/fimmu.2019.01688

**Published:** 2019-07-17

**Authors:** Bin Wan, Emilie Goguet, Marc Ravallec, Olivier Pierre, Séverine Lemauf, Anne-Nathalie Volkoff, Jean-Luc Gatti, Marylène Poirié

**Affiliations:** ^1^Université Côte d'Azur, INRA, CNRS, ISA, Sophia Antipolis, France; ^2^INRA, Univ. Montpellier, UMR 1333 “Microorganism and Insect Diversity, Genomes and Interactions” (DGIMI), Montpellier, France

**Keywords:** *Drosophila*, immunity, parasitoid wasp, Leptopilina, venosomes, lamellocyte, virulence

## Abstract

Endoparasitoid wasps, which lay eggs inside the bodies of other insects, use various strategies to protect their offspring from the host immune response. The hymenopteran species of the genus *Leptopilina*, parasites of *Drosophila*, rely on the injection of a venom which contains proteins and peculiar vesicles (hereafter venosomes). We show here that the injection of purified *L. boulardi* venosomes is sufficient to impair the function of the *Drosophila melanogaster* lamellocytes, a hemocyte type specialized in the defense against wasp eggs, and thus the parasitic success of the wasp. These venosomes seem to have a unique extracellular biogenesis in the wasp venom apparatus where they acquire specific secreted proteins/virulence factors and act as a transport system to deliver these compounds into host lamellocytes. The level of venosomes entry into lamellocytes of different *Drosophila* species was correlated with the rate of parasitism success of the wasp, suggesting that this venosome-cell interaction may represent a new evolutionary level of host-parasitoid specificity.

## Introduction

It is now well-established that cells can communicate at “long distance” using diverse types of membranous particles, called extracellular vesicles (EVs). The release or secretion of EVs is a universal cellular mechanism, shared by archaea, bacteria, and eukaryotes, which probably existed in their last common ancestor (1, 2). Since most organisms interact with each other, one species can also transfer EVs to a different species and thus act on it via that specific interaction. Accordingly, recent data from bacteria, intracellular parasites, and nematodes, have shown that EVs secreted by pathogens participate in the communication and disease transmission between non-infected and infected host cells (3, 4). EVs may play a key role in altering the function of targeted cells during infection, through the spreading of pathogens or the transport of virulence factors (5, 6).

The lifestyle of the hymenopteran endoparasitoid wasps is between those of parasites and predators: they lay eggs in or on the body of other insects, their larvae develop by consuming living host tissues, resulting in the host death (7). To ensure the successful development of eggs and larvae, parasitoids have developed various mechanisms allowing to bypass the host immune response and regulate its physiology (7, 8). They have notably evolved astonishing strategies for delivering virulence factors into host cells. The production of EVs in parasitoid wasps has been known for a long time and data have revealed their various nature and origin (9–12). Some species use viral genes stably integrated in the wasp genome to build specific vesicles [named polynaviruses (PDVs) or virus-like-particles (VLPs) (9–11)]. These EVs are produced and secreted by the cells of the wasp ovarian calyx, and carry either wasp DNA (PDVs) or proteins (VLPs). Others use a more peculiar means, they produce virulence factors in the venom gland and package them in vesicles stored in the reservoir (both parts of the reproductive tract) (9, 12, 13). In all cases, these parasitoids inject EVs along with the egg during oviposition, which participate in the reproductive success of the wasp. While many parasitoid wasp species produce ovarian EVs, very few described species produce EVs in the venom apparatus (9). Among them are the wasps of the Figitidae family, including the genera *Ganaspis* and *Leptopilina* that parasitize *Drosophila melanogaster* (Diptera) and other closely related species. The venomous EVs of these species differ in shape, size, and structure (12–19), and former publications called them Virus Like Particles (VLPs) because the mature vesicles somehow resembled viruses, particularly those of *L. heterotoma* that showed spikes extending from a round/ovoid vesicle (9, 18). Recently, a proteomic study of *L. heterotoma* VLPs showed that they contain many different proteins. Based on some homology they observed between certain of these proteins and bacterial proteins, the authors proposed to rename the VLPs, mixed strategy extracellular vesicles (MSEV) (19). However, since none of the studies to date have provided indisputable evidence of a viral or bacterial origin of *Leptopilina* VLPs, we will thereafter refer to these venom vesicles with the neutral term “venosomes.” This will also avoid any confusion with the vesicles produced in the ovaries of some wasps that truly derive from a viral machinery, also named VLPs (9).

The main defense of insects against parasitic eggs and larvae, called encapsulation, involves the formation of hemocyte layers around the foreign body as well as the production, via the activation of the phenoloxidase (PO) cascade, of melanin and cytotoxic radicals presumed to kill the parasite (20). This wasp-induced reaction is largely used as a model to study the innate immune response and stimulate hematopoiesis in *Drosophila* hosts (21). Indeed, in *D. melanogaster* larva, the process of encapsulation requires the production of different types of hemocyte, including the lamellocytes that are encapsulation-specific cells induced only in case of parasitism (8, 20, 21). Lamellocytes have been identified as targets of *L. heterotoma* purified venosomes. Mixed *in vitro* with these cells, they induce their lysis, a phenomenon also observed after parasitism (12, 22) but whose precise mechanism of action remains unknown.

The parasitism success of different *Leptopilina boulardi* strains from different laboratories differs according to the *Drosophila* species or strain. For instance, two strains with different phenotypes have been characterized, ISm (strain G431, Immune Suppression for *D. melanogaster*), highly virulent against *D. melanogaster*, and ISy (strain G486, Immune Suppression for *D. yakuba*) whose success depends on the resistant/susceptible genotype of the host (23, 24). These strains differ in the relative abundance of their major venom components, with few common abundant proteins and significant quantitative differences for most of them (25). They apparently differ as well in the venosomes present in their reservoir (13). Moreover, one of the proteins in the venom of ISm that seems important for parasitism success is a Rho GTPase activating protein (RhoGAP) named LbGAP (26, 27). LbGAP belongs to a family of genes specifically expressed in the venom glands of both ISm and ISy. ISm LbGAP has been immunolocalized in the lamellocytes of the host after parasitism and the number of LbGAP spots is correlated with the degree of alteration of the lamellocyte morphology (passage from round flat to bipolar), a change supposed to affect their ability to encapsulate the parasitoid egg (28). However, the mechanisms of entry and action of LbGAP into lamellocytes remain unknown. Although the ISy strain venom also contains Rho GAPs, its success mainly relies on a serine protease inhibitor (a serpin called LbSPNy) that inhibits the PO cascade activation in the hemolymph of *Drosophila yakuba* larva, one of its main hosts (29). The LbSPNm allelic serpin that targets different types of proteases from those targeted by LbSPNy is also abundant in the venom of ISm (25).

Based on these previous data, our goal was to clarify the connection between the venom factors described and the venosomes in *L. boulardi* wasps and to study the potential link between these extracellular vesicles and the transport of such factors into host lamellocytes. We demonstrate here that venosomes may have an atypical extracellular biogenesis in the venom gland and that purified venosomes from the venom reservoir are sufficient to mimic the protective effect of the whole venom for the wasp egg. Using fluorescently labeled purified venosomes and co-immunolocalization, we showed that they target the *D. melanogaster* lamellocytes and serve as a specific transport system to deliver venom factors both to the circulating and sessile hemocytes/lamellocytes. Finally, we observed that the level of venosomes entering into lamellocytes is correlated with the success of wasp parasitism on the *Drosophila* species tested, suggesting a key role in the specificity of the host-parasitoid interaction. The parasitoid wasp *L. boulardi* thus appears as an interesting model to study the evolution of extracellular vesicle formation mechanisms and the role played by the vesicular transport in interspecies communication. This new cellular level of parasitoid specificity may also be of interest for understanding the adaptive mechanisms between hosts and parasites.

## Materials and Methods

### Biological Material

The *L. boulardi* strains ISy from Brazzaville (Congo) (Gif stock G486) and ISm from Nasrallah (Tunisia) (Gif stock G431), and the *L. heterotoma* strain from Gotheron (France) have been previously described (23–25). All parasitoids were reared at 25°C on the susceptible *D. melanogaster* strain Nasrallah (Gif stock 1333). After emergence, adults were kept at 20°C on agar medium with honey. All experiments were performed with naïve, 5–10 days-old mated females.

The *D. melanogaster* strain 1088, named YR for resistant to the *L. boulardi* ISy parasitism, comes from an original selection of isofemale lines obtained from a population of Brazzaville (Congo), combined with subsequent genetic approaches (30–32). *D. melanogaster* hop^*Tum*−*l*^ (stock 8492) was obtained from the Bloomington *Drosophila* stock center. The *D. suzukii* strain, kindly provided by Dr. R. Allemand (LBBE, University Lyon 1, France), originates from a population collected in Sainte-Foy-lès-Lyon (Rhône, France). The *D. yakuba* R strain (stock number 307-14) and the *D. simulans* Japanese strain were kindly provided by D. Joly (EGCE, Gif-sur-Yvette, France) and M.T. Kimura (Hokkaido University, Japan), respectively. Based on previous laboratory results (see also Discussion), the *L. boulardi* ISm strain is highly successful on *D. melanogaster* YR and hop^*Tum*−*l*^ strains (>90%), less on *D. simulans* (>60% success of parasitism), and it consistently fails on *D. yakuba* and *D. suzukii* (<10 and 0% success of parasitism, respectively). All *Drosophila* were reared on a standard medium (10% cornmeal, 10% yeast, agar, and Nipagine) at 25°C. Conditions for all insects were a 12/12 h light/dark and 50% humidity.

### Antibodies

We used “in house” polyclonal rabbit antibodies directed against full-length recombinant proteins for LbGAP (25) and LbGAP2 (33), and synthetic peptides for LbSPN (34) and Atilla, a *D. melanogaster* marker of lamellocytes (35). The anti-LbGAP, anti-LbGAP2, and anti-LbSPN antibodies recognize the ISm and ISy proteins equally. For Western blots, we used them at dilutions 1/10,000 (LbGAP), 1/5,000 (LbGAP2), 1/10,000 (LbSPN), and 1/1,000 (Atilla). LbGAP, LbGAP2, and LbSPN antibodies were all used at a 1/500 dilution for histoimmunochemistry. Secondary antibodies were goat anti-rabbit horseradish peroxidase conjugated (1/10,000, Sigma) for Western blot, and fluorescently labeled goat anti-rabbit IgG (Fluoprobes 594, Interchim; 1/200, and 1/2,000 as indicated) for immunohistochemistry.

### Venom Recovery and Purification of Venosomes

The wasp venom apparatus was obtained by traction on the female ovipositor, the reservoir was separated from the glands and dilacerated with tweezers in 20–50 μl drop (1 reservoir per μl; number depending upon the experiment) of Insect Ringer Solution (IR; KCl, 182 mM; NaCl, 46 mM; CaCl_2_, 3 mM; Tris-HCl, 10 mM) supplemented with a protease inhibitor cocktail (IR-PI) (Sigma). This venom total extract was then centrifuged for 5 min at 500 g to remove residual tissues and obtain the “crude” venom. Crude venom was centrifuged at 15,000 g (15 min; 4°C) to pellet the vesicular material, which was washed twice with IR-PI and used for either SDS-PAGE, labeling, or electron microscopy. The supernatant was the soluble venom proteins fraction. The protein profile of the venosomes obtained by direct centrifugation was compared with that obtained by ultracentrifugation (35,000 g, 1 h; MLA50 rotor, Beckman) on a 10–50% Nycodenz gradient as previously described (12).

### SDS-PAGE and Western Blotting

Samples were run under denaturing and reducing (5% ß-mercaptoethanol) conditions on 12.5% polyacrylamide gels. The gels were either stained silver or transferred to a nitrocellulose membrane (Millipore). The membrane was blocked with TBS-Tween, 2% low fat milk, incubated overnight at 4°C with the indicated antibody, and washed and incubated with the goat anti-rabbit HRP secondary antibody for 2 h at RT. After washing, a signal was detected with a chemiluminescent substrate (Luminata Western, Millipore) with a digital camera. None of the preimmune sera or secondary antibody alone produced a significant signal.

### Electron Microscopy

15,000 g venosome pellets were processed for transmission electron microscopy as described in Labrosse et al. (14). Briefly, samples were fixed in sodium cacodylate (0.1M, pH 7.2 for venosomes) or PBS (for the venom apparatus) with 5% glutaraldehyde for 24 h at 4°C. Post-fixation was done with 2% osmium tetroxide in the same buffers, followed by dehydration in a graded ethanol series prior to inclusion in Epon and ultrafine section. The sections were contrasted with uranyl acetate and lead citrate before observation (Jeol 1010 and Zeiss EM10CR, 80 kV).

For immunogold labeling, 24 venom apparatus (gland and reservoir) from ISm and ISy females were fixed for 3 h in 4% paraformaldehyde −0.1 M phosphate at 4°C. Samples were included in agarose blocks (8 apparatus per inclusion; three blocks for each strain). The blocks were washed four times in 0.1 M phosphate buffer at RT and dehydrated in ethanol (50–100% at −19°C), before inclusion in the resin (London Resin White; TAAB Lab Equipment; −25°C for 48 h under UV). Ultrafine sections (70 nm) were mounted on grids treated with colloidal gold, washed in PBS, and blocked in PBS-1% BSA before incubation with the LbGAP antibody (1/1,000; 1 h 30 at 20°C) (this last step was omitted for controls). After several washes in PBS-1% BSA and then in PBS-0.1% BSA, all the grids were incubated with a goat anti-rabbit antibody conjugated to 15-nm gold particles (1/30; Biocell Research Lab.). After washing, sections were stained with 1% uranyl acetate and examined under an electron microscope (JEOL 1010 at 80 kV). All observations (controls and processed) were performed on successive sections of a block for each wasp strain. Sections from several blocks were observed and photograph.

### Microinjection

The venosome pellet obtained from 20 reservoirs was resuspended in 20 μl of IR and labeled with 1 mM of Fluoprobes 488-NHS ester (1 mg/ml; λexc./λem.: 593/519 nm; Interchim) for 1 h at 4°C. The labeled vesicles were centrifuged at 15,000 g (10 min at 4°C) and washed once with IR-3% BSA to quench the free ester, and again with IR. The last pellet was resuspended in IR and used to micro-inject 50 second-instar host larvae (FemtoJet, Eppendorf). Hemolymph was collected 14–18h after microinjection.

To test the phagocytic properties of the hemocytes *in vivo*, fluorescent latex beads (carboxylate-modified polystyrene latex beads 2 μm in diameter, fluorescent red; Sigma) or fluorescent live *Escherichia coli* (5 ×10^9^ bacteria/ml; *E. coli* DH5 alpha expressing the green fluorescent protein) were microinjected in *Drosophila* L2 larvae 14–18 h after parasitism (to stimulate the hemocytes production). The observations were made 4 h after the injection of beads or bacteria. The experiments were repeated 3 times for each species tested, on different days.

### Parasitism Assay

For immunohistochemistry of hemocytes, batches of 30 second-instar larvae (L2) were collected and transferred on a dish with *Drosophila* medium to be subjected to parasitism by three female wasps for 4 h. The parasitoids were then removed, and the larvae were left at 25°C until use (see below). For the parasitism experiments, 30 second-instar host larvae (L2) were parasitized for 2 h by one ISm or ISy parasitoid female. The encapsulation capacity was estimated 48 h later by counting the number of encapsulated eggs after dissection of the late third-instar larvae. Virulence was expressed as the ratio of the number of non-encapsulated parasitoid eggs to that of the mono-parasitized hosts. To analyze the immunosuppressive role of the different fractions of ISm venom (crude venom, 15,000 g venom pellet, 15,000 g venom supernatant), larvae of *D. melanogaster* YR were injected with 20 nl of each of these fractions using a Nanoject II injector (Drummond Scientific). The samples were obtained from 20 female reservoirs diluted in 20 μl of IR, this volume being kept constant so that a 1/50th equivalent of a reservoir was injected for each fraction. A total of 150 larvae (5 independent repeats of 30 larvae) were injected for each sample, then parasitized by ISy female parasitoids, and encapsulation occurred an estimated 48 h later on surviving larvae, as described above. The controls were injected with IR alone.

### Hemolymph and Hemocyte Collection

*Drosophila* larvae were removed from the food and washed carefully three times in PBS. Their hemolymph was then collected directly in a drop of 35 μl of PBS (SDS-PAGE) or Grace's medium (Immunochemistry) by gently tearing the cuticle on the anterior part of the larvae with fine tweezers. For SDS-PAGE, the cells were separated by centrifugation (500 g, 10 min) and the supernatant was collected (cleared hemolymph). The cells pellet was washed twice in PBS and the last pellet and the cleared hemolymph were diluted in reducing sample buffer and boiled for SDS-PAGE. At least three separate experiments were done.

### Tissue Immunohistochemistry and Confocal Observation

#### Hemocyte Immunohistochemistry

The hemolymph of *Drosophila* larvae was collected at various time points (as indicated) after parasitism or micro-injection with labeled venosomes. At each time point, the hemolymph was collected from 10 larvae as described above, and the hemolymph solution was transferred to the center of a coverslip placed in a 12-well culture plate to form a wet chamber. The cells were allowed to adhere for 1 h before a 15 min fixation step with 4% formaldehyde in PBS. The cells were then washed three times with PBS, permeated with PBS-T (PBS + 0.1% Triton 100X) for 15 min and blocked with PBS 0.3% BSA for 30 min. The cells were incubated for 1 h with a primary antibody at RT, washed three times with PBS and incubated for 1 h with the secondary antibody. After three washes with PBS and one with deionized water, the coverslip was mounted on a slide using an antifading medium containing DAPI (Interchim). Data were obtained from at least three separate experiments.

#### Long Gland Immunohistochemistry

Complete venom apparatuses were fixed in 4% formaldehyde 0.1% Triton 100X for 3 h. After that, they were washed three times with PBS and blocked with PBS 0.3% BSA for 30 min. The primary antibody was added and incubated for 1 h, washed three times 15 min with PBS, and incubated with the secondary antibody for 1 h at RT. After three PBS washes, venom apparatuses were mounted between a slide and a coverslip, as described above. 5 apparatuses were treated per experiment and the experiment was repeated at least four times.

For both types of preparation, the actin was stained with green phalloidin (7 nM; green fluorescent phalloidin 490, Interchim) during the permeabilization step. Controls were done with the pre-immune sera from the rabbit used for immunization and with the secondary antibody alone. The highest background signal obtained was used to establish the level above which a labeling was considered positive. The samples were observed and imaged with an AxioImager Z1 equipped with an Apotome 2 or with a LSM 880 laser scanning confocal microscope (Zeiss).

### Clarification and Observation of *Drosophila* Larvae

YR larvae were fixed overnight, 15 h after injection of fluorescently labeled venosomes, with 4% PFA in PBS at 4°C, in the dark. After washing with PBS, the samples were transferred to a glass vial for a clearing procedure based on the 3DISCO method (36). Briefly, the samples were dehydrated overnight in 50% Tetrahydrofurane (THF) (Sigma) in the dark while being gently shaken, then for 3 h in 70% THF, overnight at 80% THF and for 30 min at 100% THF, repeated 3 times, followed by final incubation in dichloromethane (Sigma) for 45 min. To ensure optimum transparency, samples were finally impregnated overnight with a dibenzylether (DBE) solution (Sigma). To avoid tissue damage or shrinkage, they were mounted in a Lab-Tek II (Lab-Tek) chamber filled with the DBE solution. As DBE degrades fluorescence over time, the visualization was made as soon as the appropriate transparency level was reached. To obtain high resolution scans, samples were imaged by confocal microscopy. 3–5 larvae were treated in three different batches.

### Statistical Analysis

Statistics were done using R (https://www.r-project.org). For the number of lamellocyte cells labeled with LbGAP in the different species, we used a binomial GLMM [package lme4 (37)] with the species as a fixed effect and the repetition as a random effect. Since there was no overdispersal in the model, the fixed effect was tested with an LRT-test, followed by a Tukey *post-hoc* test [package multcomp (38)]. For the number of spots in the lamellocytes, we first used a zero truncated Poisson GLM [VGAM package (39)] with the species as a fixed effect. However, since there was a high dispersal in this model, we instead fitted a linear model to the Box-cox transformed number of lamellocyte cells, followed by a Tukey *post-hoc* test.

## Results

### LbGAP and LbGAP2 Association With *L. boulardi* Venosomes

**Figure 1:**
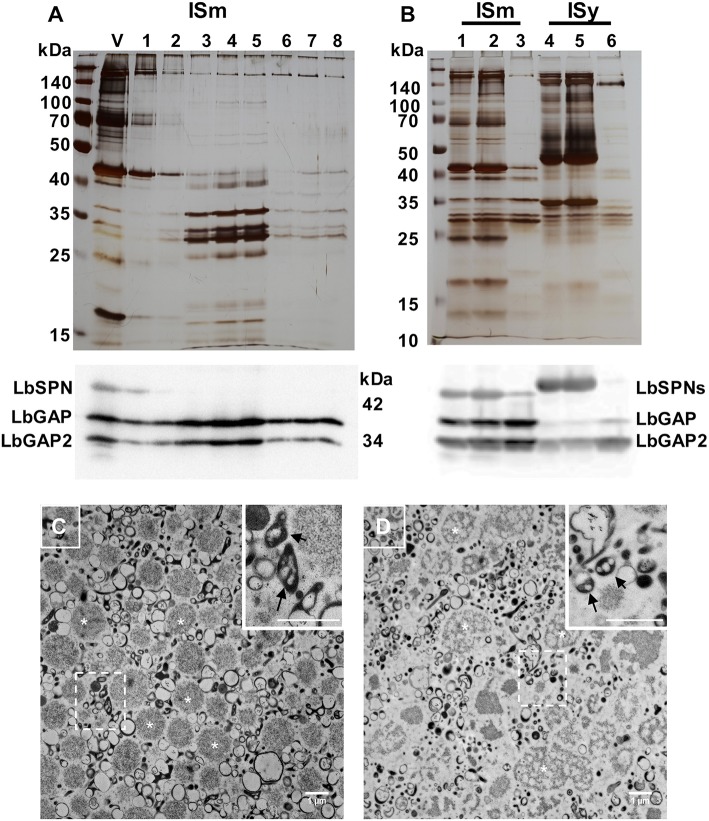
*L. boulardi* ISm and ISy venosomes are enriched in LbGAP and LbGAP2. **(A)** Crude ISm venom (V) was ultra-centrifuged on a 10–50% Nicodenz^®^ gradient, and each of the recovered fractions (1 top to 8 bottom) were separated on a 12% SDS-PAGE (upper figure). The venosomes migrated on fractions 3–5 in which the ~30 kDa LbGAP and LbGAP2 proteins were enriched but not LbSPN (~45 kDa) as shown by the western blot analysis (lower figure). **(B)** Proteins in ISm and ISy crude venom (lanes 1 and 4, respectively), 15,000 g supernatant (lanes 2 and 5) and pellet (lanes 3 and 6) separated by SDS-PAGE and visualized by silver-staining (upper figure) or after western blot with the anti-LbGAP, LbGAP2, and LbSPN antibodies (lower figure). In both cases, the protein complexity is reduced in the 15,000 g pellet and the LbGAP and LbGAP2 proteins are enriched while the LbSPN proteins remain in the supernatant as shown on the western blots. **(C,D)** TEM of fixed ISm **(C)** and ISy **(D)** 15,000 g pellets showing diverse types of vesicles and aggregates including the previously described venosomes [indicated by arrows in the insert showing the enlargement from dashed square zone (Bar = 1 μm)]. Examples of the large vesicles/aggregates type (in the 1 μm range) are indicated by white asterisk.

The venom apparatus of *L. boulardi* consists of an elongated venom gland (classically named “long gland”) with a central collecting canal connected by a thin duct (connecting duct) to a large reservoir in which venom accumulates (see detailed histology of the venom apparatus in Figure S1). To determine whether the LbGAP, LbGAP2 and LbSPN *L. boulardi* venom proteins potentially involved in virulence were associated with venosomes, we first purified the venosomes from crude venom obtained from the ISm reservoir using the previously described method of Nycodenz gradient ultracentrifugation (12) (Figure 1A). SDS-PAGE of the protein fractions showed that only a specific subset of the venom proteins co-sedimented at the expected position of venosomes in the gradient (fractions 4, about 25–30% Nicodenz). Western blot analysis revealed that LbGAP and LbGAP2, but not LbSPN, were associated with these venosome fractions. In order to confirm this, as well as to simplify and down-size the purification procedure, the crude ISm and ISy venom was directly centrifuged at 15,000 g. The protein profile of the washed pellet contained only a subset of the total proteins, very similar to that of Nycodenz-purified ISm venosomes fractions. Western blot analysis showed that LbGAP and LbGAP2 were enriched in the pellet but not LbSPN, which remained in the supernatant along with many other proteins (Figure 1B). Electron microscopy analysis of the pellet showed two main types of vesicles: large round aggregates/vesicles with a size of about 1 μm (asterisk marks; Figures 1C,D) and, in the range of 100–300 nm, “typical” venosomes (arrows in enlargements in Figures 1C,D) and vesicles with different shapes that were less numerous and structured in ISy than ISm (Figures 1C,D). Because the shape of most of these purified vesicles at 100–300 nm appeared to differ from that of the previously “typical” venosomes described in the venom reservoir of *L. boulardi* strains (13, 14), we verified that the procedure did not extensively degrade the venosomes. Since *L. heterotoma* venosomes, in contrast to *L. boulardi* ones, have a specific stellate shape due to spike extensions (12, 17), we treated the crude venom of this species in the same way and analyzed the obtained pellet by microscopy (Figure S2). Part of the *L. heterotoma* pelleted vesicles clearly retained the stellate shape, while others resembled more *L. boulardi* ISm and ISy vesicles, with the electron dense material accumulated as an asymmetric crescent or distributed all along the membrane [Figure S2; see also Figure 1E in (12)]. This suggests that the treatment may have slightly altered the shape of the venosomes but that the distinctive shapes between the different *Leptopilina* species and strains remained visible.

**Figure 2:**
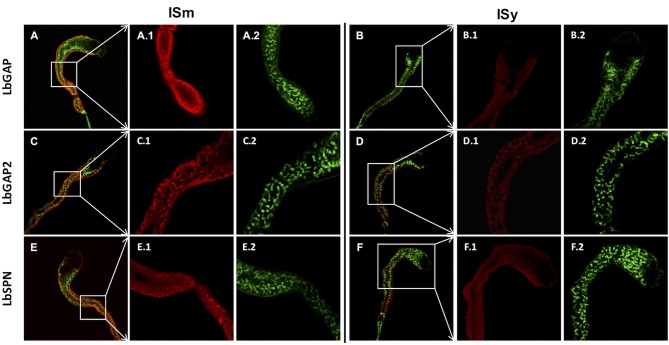
Immunolocalization of venom virulence factors along the venom gland of ISm and ISy females. Confocal immunolocalization of LbGAP **(A,B)**, LbGAP2 **(C,D)**, and LbSPN **(E,F)** in the ISm **(A,C,E)** and ISy **(B,D,F)** venom gland. **(A–F)** Show merged images obtained from the entire gland for the indicated virulence factors (red fluorescence) and counter-stained for actin with green phalloidin. All panels numbered 1 and 2 are enlargement of the indicated gland region (white boxes) for the virulence factors (red) and actin (green), respectively. Labeling of the three virulence factors was observed in the cytoplasm of the peripheral gland secretory cells and associated with the canal of these cells whose microvilli actin was strongly labeled by phalloidin. For both strains, the absence of labeling in the gland collecting canal is either due to the loss of the contents during the staining process or to a masking due to the presence of cuticular layers that may block the light.

**Figure 3:**
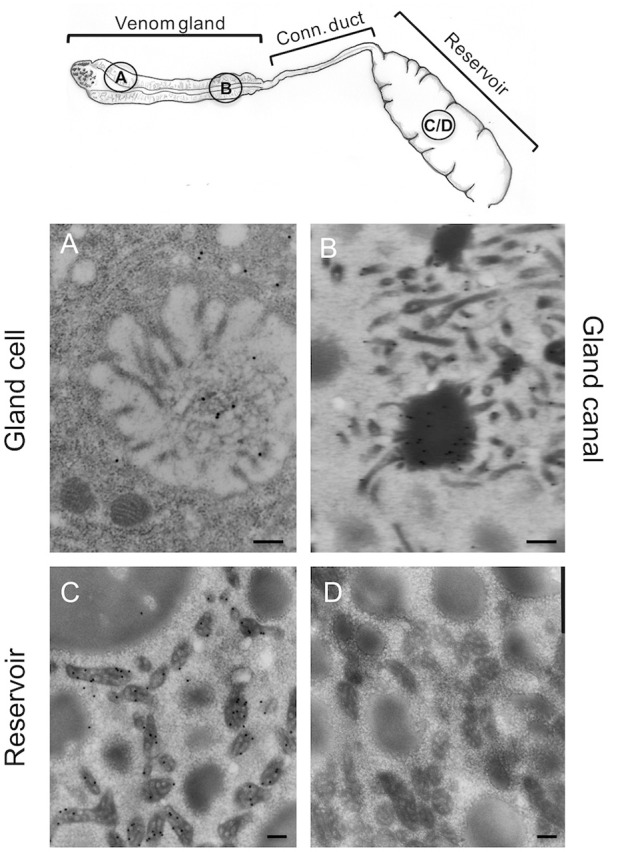
Immunolocalization of LbGAP during the biogenesis of ISm venosomes. Immunogold-labeled TEM sections (see drawing at the top for the position of the sections) through the intracellular canal of a long gland secreting cell **(A)**, the gland canal **(B)**, and the reservoir **(C,D)**. LbGAP immunogold labeling (small black dots) is visible on the amorphic material present in the lumen of secretory cell canal, around in the cytoplasm of the cell and in some intracellular vesicles **(A)**. No secreted membrane vesicles were observed. In the lumen of the gland collecting canal **(B)**, most of the labeling was associated to electron-dense aggregated and fibrous material. No clear fully formed vesicles were visible. In the reservoir **(C)**, gold labeling was almost entirely associated with the mature punctuated venosomes but not with the larger electron dense particles also present. No labeling was observed with the gold secondary antibody alone **(D)**. Representative sections are shown here. Bar 0.2 μm.

**Figure 4:**
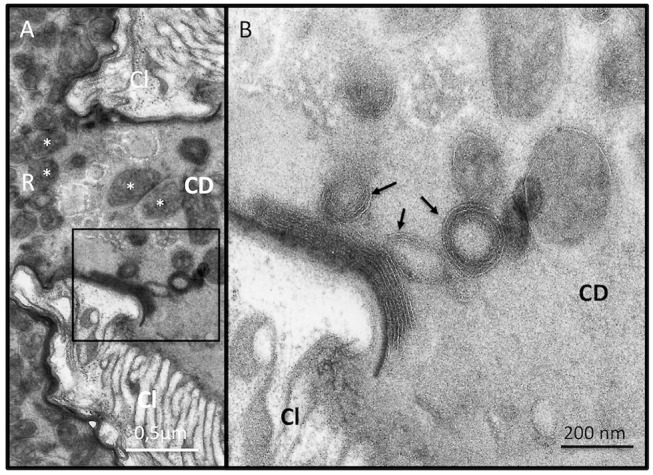
Biogenesis of the membrane of extracellular venosomes. **(A)** TEM observation of a section through the junction between the connecting duct (CD) and the reservoir (R) and an enlargement of the boxed zone **(B)** show stacks of membrane sticking to the lumen side of the cell layer (Cl) located at the junction. Some “rolling” empty membranes detach from the stack and form multimembrane vesicular structures (arrows) incorporating material present in the duct lumen. Punctuated material could also be observed in formed venosomes (in **A**, white asterisk) that are accumulated in the reservoir (R).

Since ISm and ISy venosomes were associated with a subset of venom proteins such as LbGAP and LbGAP2 but not with LbSPN, we wondered whether the synthesis and secretion of these factors (and thus the biogenesis of venosomes) could occur sequentially at different or specialized locations along the venom gland. LbGAP, LbGAP2, and LbSPN antibodies (Figure 2) labeled the whole ISm (Figures 2A,C,E, respectively) and ISy (Figures 2B,D,F, respectively) gland (except the “nose” region), although the signal was much weaker for LbGAP in ISy (Figure 2B), in agreement with a lower synthesis of this protein in this strain (26, 27) (see also Figure 1). The factors were clearly immunolocalized in the secretory cell cytoplasm and in the lumen of these cells' channels (as shown in the respective Figures enlargements) that can be visualized by the phalloidin labeling of actin-rich microvilli as described previously in *L. heterotoma* (17) (see also Figure S3 for a detailed example of co-localization for LbGAP2 in the ISm cell and secretory cell channel). Therefore, it seems that there is no difference in secretion along the gland between the factors associated and not associated with venosomes. The venosomes of the Figitidae wasps were previously described as having a peculiar assembly in the lumen of the venom gland (15–18), and we therefore studied further how LbGAP could associate with the ISm venosomes by electron microscopy. For this, ultra-thin sections of the ISm venom apparatus were prepared for immunogold electron microscopy using the LbGAP antibody (Figure 3). Figure 3A show a cross-section through the “canal” of the secretory cells as indicated by the microvilli presence. The canal contains large electron dense particles, without precise form, which were immunogold labeled for LbGAP (Figure 3A). In the collecting canal of the gland (Figure 3B), large electron-dense particles and long filaments were dispersed in a fibrous/filamentous material. LbGAP was almost exclusively associated with the electron dense particulate materials suggesting proteins aggregation. A cross-section through the ISm reservoir showed venosomes as membranous punctuated-type vesicles (Figures 3C,D) and larger uniform electron-dense particles (from 400 nm to 1 μm), with 87% of the LbGAP labeling associated with the venosomes (333 gold beads associated on 383 counted on three different TEM sections) (Figure 3C). One of the control cross-sections that passed at the junction between the connecting duct and the reservoir caught our attention (Figure 4). At this point, we observed a stack of membranes coming from the cell lining the duct, some of them seeming to roll and detach to form multiple membrane vesicles.

Thus, *L. boulardi* ISm venosomes seem to be built extracellularly from secreted proteins aggregates that modify their form and size along the collecting duct. Since none of the particulate aggregates observed in the ISm gland canal resembled the “mature” membraned venosomes observed in the reservoir (see Figure 3), we hypothesized that a specific part of the lumen material could be embedded by the membranes delaminating in the collecting duct near the entry of the reservoir to form the final mature venosomes.

### Venosomes Are Sufficient to Protect Susceptible Wasp Eggs From Encapsulation

Since *L. boulardi* ISm venosomes contain putative virulence factors, we tested whether these vesicles were sufficient to replicate the protective effect of venom against encapsulation of eggs of the susceptible ISy strain (14, 23). For this, we used the previously set up assay (14, 24) based on the two different characterized *L. boulardi* strains, ISm, and ISy, and the resistant *D. melanogaster* strain (YR). ISm eggs are never encapsulated in *D. melanogaster* YR larvae, whereas those of ISy almost always are (23, 24) (see Table 1). *D. melanogaster* YR larvae injected with extracts of ISm venom apparatus or crude venom and then parasitized with ISy did not encapsulate the parasitoid eggs (14, 24) (Table 1). This protective effect of the crude venom against ISy eggs encapsulation was also conferred by injection of the ISm washed 15,000 g pellet containing the venosomes (Table 1, mean % of 5 replicates; comparison to injection of ISm crude venom, χ^2^ = 0.91; *p* < 0.05). In contrast, the injection of the supernatant 15,000 g of the ISm venom had no effect (comparison to injection of Ringer, χ^2^ = 0.711; *p* < 0.05).

**Table 1:** Outcomes of parasitism after injection of crude venom, 15,000 g venosomes pellet or venom supernatant.

	**% of encapsulated parasitoid eggs**
ISm parasitism	0
ISy parasitism	94
Ringer injection	94
ISm crude venom injection	40
ISm 15,000 g supernatant injection	98
ISm 15,000 g pellet injection	24

### Venosomes Transport Potential Virulence Factors in Permissive Host Lamellocytes

LbGAP was immunolocalized in lamellocytes after parasitism (27, 28). Also, its physical association with the venosomes led us to postulate that venosomes are responsible for the transport of this protein and other associated proteins in immune cells of the host. To demonstrate this definitively, we immunolocalized LbGAP and LbGAP2 (both associated with venosomes) by confocal microscopy in the hemocytes of *D. melanogaster* YR larvae after parasitism (*in vivo* situation). We also tested by confocal microscopy the co-immunolocalization of LbGAP or LbGAP2 and venosomes in lamellocytes after microinjection of *in vitro* fluorescently-labeled purified venosomes.

**Figure 5:**
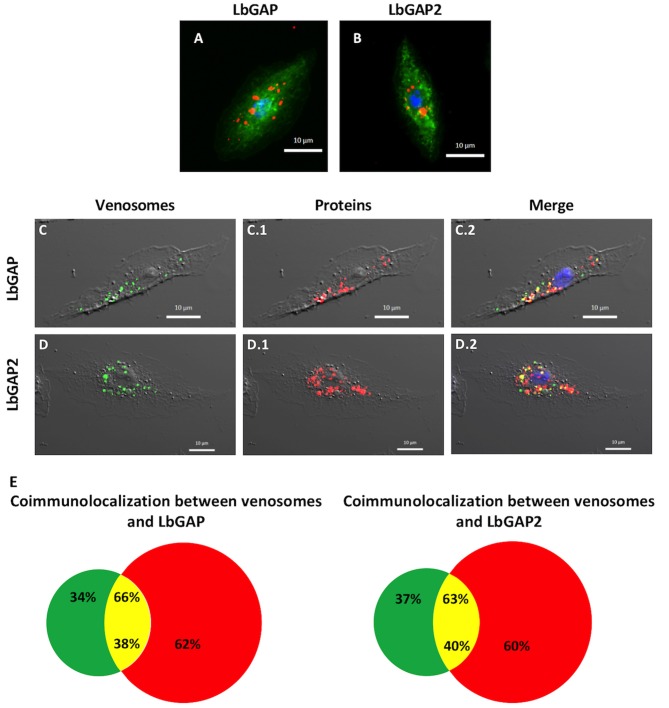
Venosomes co-immunolocalize with virulence factors in *Drosophila* lamellocytes. Immunolocalization of LbGAP **(A)** and LbGAP2 **(B)** by fluorescence microscopy (red spots) in the cytoplasm of bipolar modified lamellocytes of *Drosophila* YR larvae 14–18 h after parasitism by ISm females. Actin labeled with phalloidin (green) and nucleus with DAPI (blue). **(C,D)** Confocal microscopy of YR lamellocytes 18 h after injection of ISm showing fluorescently labeled venosomes (green spots, **C,D**), and LbGAP **(C.1)** and LbGAP2 **(D.1)** immunolocalization (red spots); On the merged images **(C.2,D.2)**, yellow spots indicate where red and green fluorescent spots co-localized. **(E)**, percentages of co-localization between venosomes and LbGAP, and venosomes and LbGAP2 were obtained from merged images (green portion, venosomes alone; red, LbGAP, or LbGAP2 alone; yellow, co-immunolocalization). The percentage of co-localization is expressed either on total venosomes spots (% on top) or on total spots of LbGAP or LbGAP2 (% on bottom). Counted on 61 and 57 cells, respectively, in two separate experiments.

**Figure 6:**
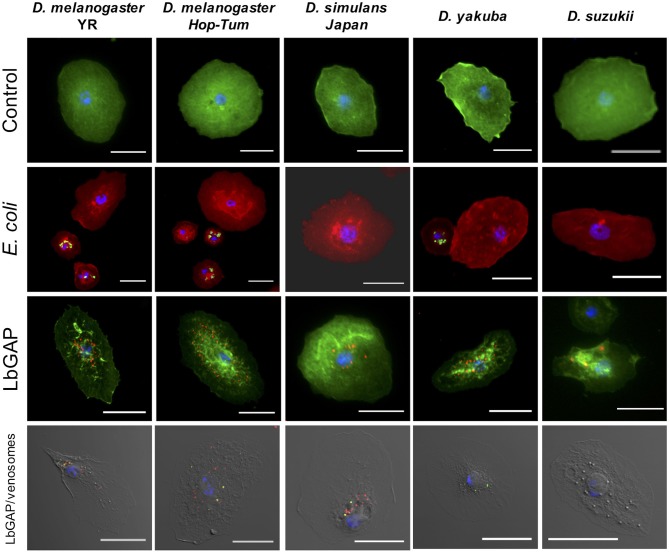
Species-specific entry of venosomes into host lamellocytes. First row: Lamellocytes in the hemolymph of *D. melanogaster* YR and hop^*Tum*−*l*^, *D. simulans, D. yakuba*, and *D. suzukii* (actin labeled with green fluorescent phalloidin, nuclei in blue with DAPI) that have retained their “normal” shape (control). Second row: lamellocytes do not contain green fluorescent *E. coli*, indicating the absence of phagocytosis, unlike plasmatocytes (actin labeled with red fluorescent phalloidin; see also Figure S6). Third row: LbGAP spots (in red) in the most reactive lamellocytes of the species tested (see also counts in Figure 7). Fourth row: the same quantity of ISm labeled venosomes were injected in L2 larvae from the different species. Eighteen hours after injection, venosomes (green spots), LbGAP (red spots), and co-localization of both (yellow spots) were observed by confocal microscopy depending upon the species (none in *D. suzukii*). Bars = 20 μm.

Between 0–4 h after the end of ISm parasitism (time laps due to uncertainty since parasitism can occur at any time during the 4 h of contact between wasps and larvae; see mat. and meth.), LbGAP and LbGAP2 mostly immunolocalized in phagocytic plasmatocytes (see also below) since, as expected, there were still no lamellocytes at this time (40). This absence of lamellocytes was also demonstrated by the absence of reaction of this cell type marker Atilla on western-blots of hemocytes proteins (see Figure S4). 14–18 h post-parasitism, both LbGAP and LbGAP2 were still observed in plasmatocytes (not shown) and accumulated in the circulating host lamellocytes (Figures 5A,B), with the shape of most of these labeled lamellocytes visibly modified from round flat to elongated as previously observed (27, 28) (see unchanged lamellocytes in Figure 6). Thus, both proteins associated with venosomes were retrieved in modified lamellocytes after parasitism.

Microinjection of second-instar YR larvae with purified fluorescently-labeled ISm venosomes, induced the differentiation of lamellocytes, like parasitism (Figure S4). 18 h post-injection, fluorescent venosomes were observed in plasmatocytes (not shown) and co-immunolocalized with LbGAP or LbGAP2 in these newly formed lamellocytes (Figures 5C,D, respectively). This strongly suggests the entry of whole venosomes into the lamellocytes since the chosen NHS-fluorescent dye should mainly label membrane proteins of the venosomes. Interestingly, not all LbGAP and LbGAP2 spots co-localized with labeled venosomes in the lamellocytes (Figure 5E), suggesting that a release of these proteins from the membrane envelope occurred after the venosomes enter the cell (this also told us that these proteins were not labeled by the NHS-dye). Moreover, using hemolymph collected at different time points after parasitism (0 h being the end of the parasitism assay) and Western blot analyses, we showed that LbGAP and LbGAP2 (and thus certainly venosomes) still circulate in the cell-free hemolymph of host larvae 20 h post-parasitism by ISm or after microinjection (Figure S4). Thus, a continuous entry of venosomes from the hemolymph into the lamellocytes, followed by the release of LbGAP and LbGAP2 in the cell, may explain our observations.

Since parasitoid wasp infestation of *D. melanogaster* induces the release into the circulation of a subcuticular population of sessile hemocytes that will differentiate into lamellocytes (40), we analyzed whether venosomes could also target the hemocytes within this compartment. Eighteen hours after the injection of labeled venosomes, *Drosophila* YR larvae were fixed, chemically treated for tissue clearing (36, 41) and observed by confocal microscopy (Figure S5). Fluorescent spots were clustered in the subcuticular regions where sessile hemocytes localize (40). At higher magnification, this labeling co-localized with small (about 5–10 μm) and large flat cells (>30 μm), resembling plasmatocytes and lamellocytes, respectively.

### Entry of Venosomes Seems “Specific” to Lamellocytes of Some Species of *Drosophila*

**Figure 7:**
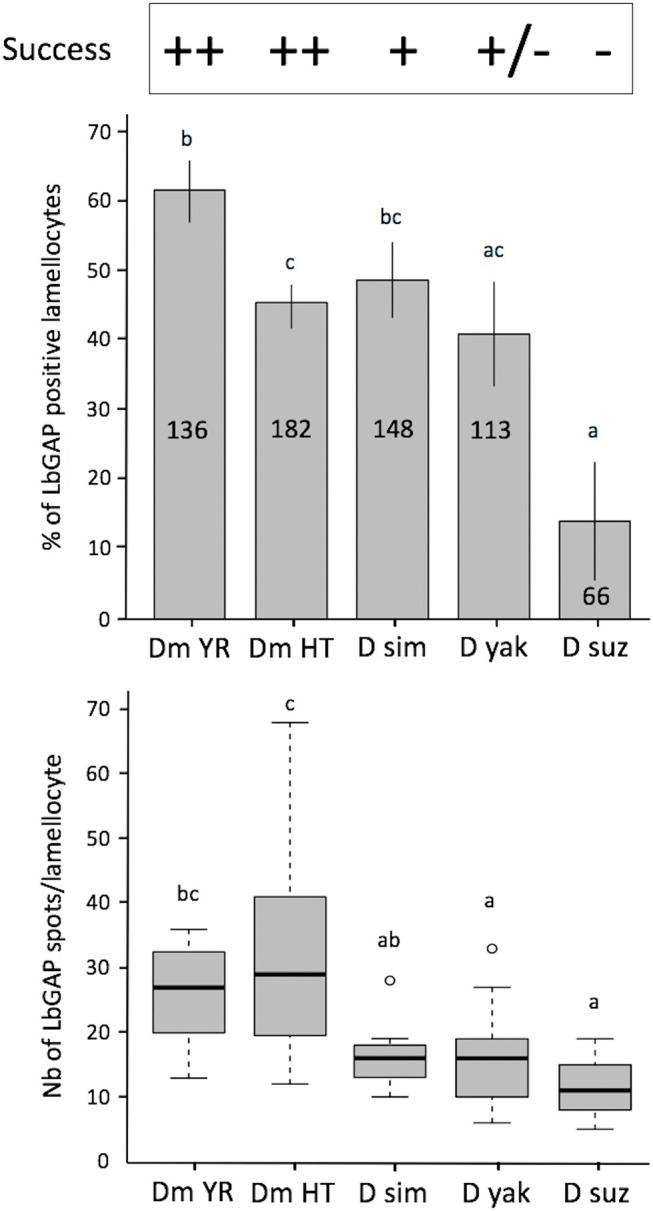
Quantification of LbGAP entry in lamellocytes of the different *Drosophila* species. On the top of the figure the parasitoid success on the different *Drosophila* species is indicated [*D. melanogaster* YR (DmYR) and hop^*Tum*−*l*^ (Dm HT), *D. simulans* (D sim), *D. yakuba* (D yak), and *D. suzukii* (D suz)] (++ > 90%; + about 60%; ± <10%; −0%). **Upper panel**: Percent of LbGAP positive lamellocytes, 18 h after parasitism by ISm females (mean ± s.d). The total number of lamellocytes counted from different fields of at least two separate experiments is indicated on the column (less lamellocytes were found in *D. suzukii* compared to the other species). **Lower panel**: number of LbGAP immunoreactive spots in the most reactive lamellocytes of the different species except for *D. suzukii* where, due to their low number, all reactive lamellocytes with more than one spot were considered (number of lamellocytes: Dm YR, *n* = 12; Dm HT, *n* = 12; D sim, *n* = 11; D yak, *n* = 13; D suz, *n* = 3) (mean ± s.d). Results from two separate experiments. The different letters indicate significant differences (*p* < 0.05).

*Leptopilina boulardi* ISm is considered a specialized parasitoid that succeeds mainly on *D. melanogaster* and *D. simulans* (23, 24). We therefore tested whether the entry of venosomes into lamellocytes depended on the host species. First, to confirm that all the *Drosophila* strains and species used produce lamellocytes, we analyzed the main adhesive hemocytes after parasitism by ISm. As a control, we used hop^*Tum*−*l*^ flies since this *D. melanogaster* mutant strain is known to constitutively produce lamellocytes (42). Two main types of cells were observed in the hemolymph of these species/strains 14–18 h after parasitism: (i) round cells of 10–20 μm diameter—resembling macrophage-like plasmatocytes or podocytes described in these species (43–46)—and having a phagocytic function against injected latex beads and *E. coli* bacteria (Figure S6), (ii) large flat cells of 30–40 μm diameter, lamellocytes (Figure 6, 1st row), which spread well on slide but do not phagocyte bacteria (Figure 6, 2nd row). After parasitism by ISm, LbGAP-containing lamellocytes were found for all species (Figure 6, 3rd row), although the number of cells that were reactive differed among species. It was significantly higher in *D. melanogaster* YR than in hop^*Tum*−*l*^ (*p* = 0.0061) and *D. yakuba* (*p* = 0.019), and lower in *D. suzukii* compared to YR (*p* < 0.001), hop^*Tum*−*l*^ (*p* = 0.043), and *D. simulans* (*p* = 0.015). *D. yakuba* thus appeared as intermediate between *D. melanogaster*/*D. simulans* and *D. suzukii* (Figure 7). The lower number of cells labeled in hop^*Tum*−*l*^ compared to YR is probably due to the very large number of lamellocytes produced by this strain. Besides, the maximum amount of LbGAP spots per reactive lamellocyte [which was previously correlated to the lamellocytes change in shape (28)] was higher in *D. melanogaster* compared to *D. yakuba* (*p* = 0.028 for YR, *p* = 0.0018 for hop^*Tum*−*l*^) and *D. suzukii* (*p* = 0.017 for YR, *p* = 0.0029 for hop^*Tum*−*l*^) as well as to *D. simulans* (only for hop^*Tum*−*l*^, *p* = 0.0075), with more than twice that found in these three last species (Figure 7). We also microinjected labeled venosomes (Figure 6, 4th row) in L2 larvae of the different species and the visual observation of the co-immunolocalization of LbGAP and venosomes indicated an analogous outcome: numerous venosomes and LbGAP spots were observed in a large number of *D. melanogaster* and *D. simulans* lamellocytes, whereas *D. yakuba* lamellocytes contained only a few labeled venosomes, but no LbGAP immunoreaction, suggesting a possible rapid degradation. After microinjection, only few lamellocytes were produced by *D. suzukii* and none were labeled. Thus, the number of labeled lamellocytes and the quantity of LbGAP/venosome they can uptake seemed to match the success rate of parasitism by ISm wasps in these *Drosophila* species [*D. melanogaster* > *D. simulans* >> *D. yakuba* and *D. suzukii* (see Discussion)]. The increased entry of venosomes into *D. melanogaster* lamellocytes may thus rely on the existence of a specific or more efficient mechanism, restricted or absent in other species.

## Discussion

Different types of vesicles are formed by Hymenoptera parasitoid wasps and injected into their hosts to ensure their reproductive success: PDVs and certain VLPs produced in the calyx cell of the wasp ovaries, derived from a viral machinery integrated in the wasp genome during evolution (9–11), and other extra-cellular vesicles types formed in the venom apparatus for a few reported species, including *Meteorus pulchricornis* (Braconidae: Euphorinae) (47), a polyphagous parasitoid of Lepidoptera larvae, and Figitidae of the genera *Ganaspis* and *Leptopilina* [the latter divided into three clades, *Heterotoma, Boulardi*, and *Longipes* (48)], which attack the larvae of *Drosophila* spp.

### Venosomes Biogenesis

Mature venosomes have already been described in the venom reservoir of *L. boulardi* ISm and ISy strains (13, 14) but their biogenesis and their associated proteins were not previously reported. The secretory cells of the wasp venom gland secrete an electron-dense anamorphic material with no apparent membrane, also present in the gland canal. For ISm, this material, reactive to LbGAP, was retrieved later specifically associated with fully formed venosomes in the reservoir. It was also only in the reservoir that the venosomes had their ovoid shape with a membrane surrounding an electron-dense material punctuated by small membranous vesicles. Venosomes were more numerous and more structured in ISm compared to ISy, whose venosomes appear to contain less dense material and fewer enclosed vesicles, as previously described in the reservoir of these strains (13). Overall, our results confirm and emphasize that the formation/maturation of venosomes in Figitidae seems to occur extracellularly during the progression of secreted material throughout the gland canal to the reservoir. The venosomes of the *Ganaspis* and *Leptopilina* species studied to date are also 100–400 nm vesicles having different shapes in the reservoir. The biogenesis of venosomes in these species occurs through various stages of aggregation from precursor materials secreted in the lumen of the gland, whereas in the reservoir, venosomes have a pentagonal/hexagonal core with multiple extension or spikes and a distinct membrane [(17, 18); this work]. Those of the *L. boulardi* Lb17 strain differed in shape and biogenesis: small unshaped vesicles were present in the canal of the secretory cells of the gland and referred to as “immature,” whereas the venosomes in the mature reservoir were larger, rounded vesicles enclosing a large amount of punctuate/vesicular material resembling those of ISm (18). The authors suggested that venosomes in the reservoir resulted from the maturation of the unshaped vesicles during the transit (18). How venom gland secreted proteins are associated/embedded in venosomes also has not been thoroughly explored. The P40 polyclonal antibody, directed against a protein of *L. heterotoma* venosomes, reacted throughout the gland, and localized with the secreted materials/blocks that assemble to form the final venosomes in which it is associated with the surface and the spikes (16–18). Here, we showed that LbGAP had a similar secretion/embedding fate during the transit. Since venosomes contain a large number of proteins [(19); see also Figure 1] and we only have observations for two of them, it is difficult to identify by which mechanism(s) the secreted material will be integrated in or will be part of mature venosomes. Besides, we showed here that only some of the venom factors studied were associated with vesicles (LbGAP and LbGAP2) while others remained soluble (LbSPN), although they were all secreted throughout the gland and quantitatively important in the venom. This suggests that this process is not a simple progressive maturation of pre-existing vesicles along the cell or canals of the gland, but involves a specific mechanism of association. Our observation of extracellular membrane stacks at the junction between the connecting duct and the reservoir in the ISm strain led us to propose that the inclusion of a specific material and the final formation of the vesicles may occur at this place. This peculiar mode of extracellular vesicle formation, very different from the classical secretion of cellular exosomes or microvesicles (1–3) and from the intracellular mechanism of formation of PDVs and VLPs (10, 11), will deserve further investigation for definitive confirmation and elucidation. One may also wonder about the specific packaging mechanism of only certain secreted proteins in the venosomes. Subsequent studies are needed to determine whether the unincorporated proteins present in the luminal fluids are in a form (i.e., non-aggregated form) different from those apparently packaged. Alternatively, the different proteins may have specific sequence patterns (possibly unknown) or modification(s) to consider. The RhoGAPs sequences, composed mainly of the RhoGAP domain, have no particular motif identified so far but possible post-translational modifications of the packaged proteins have not yet been analyzed.

### Role of Venosomes

Purified VLPs of *L. heterotoma* induce lamellocyte lysis of hop^*Tum*−*l*^ larvae *in vitro*, and TEM observation suggested that these vesicles may enter the cytoplasm of these cells (12, 16, 22, 49). The association of the P40/P42 protein with *L. heterotoma*/*L. victoriae* venosomes and its immunolocalization in lamellocytes incubated with the venom of the reservoir led to postulate that the venosomes deliver associated proteins or toxins to lamellocytes (16, 19, 22). Based on our previous observations that LbGAP immunolocalized as “large spots” in *D. melanogaster* lamellocytes (27), we also suggested the association of LbGAP with *L. boulardi* venosomes, thus facilitating its entry into these cells. Here, the role of *L. boulardi* ISm venosomes in the active immune protection of the wasp egg has been clearly demonstrated by (i) the protection of *L. boulardi* ISy eggs from encapsulation by YR flies provided by the injection of purified ISm venosomes, (ii) immunolocalization of LbGAP and LbGAP2 in host lamellocytes whose shape was modified after ISm parasitism or microinjection of purified venosomes, and (iii) co-localization of these putative virulence factors in host modified lamellocytes together with *in vitro* fluorescently-labeled venosomes microinjected into the host. Since about 60% of LbGAP or LbGAP2 was not associated with venosomes in this last experiment, they may have already been released within the cell compartments, with the mechanism and timing of delivery still to be identified. Interestingly, although part of the circulating venosomes may be removed from the circulation by the phagocytes, the presence of free venosomes in the hemolymph seems to last long enough to continuously block newly formed lamellocytes, thus weakening the immune system of the larvae and disrupting encapsulation, and protecting also newly hatched parasitoid larvae. Moreover, *L. boulardi* venosomes appear to target not only circulating hemocytes, but also sessile hemocytes in *D. melanogaster*, suggesting that parasitism may also impair their functions. Thus, whatever their shape or the presence or absence of spikes, venosomes enter host lamellocytes. We can therefore conclude that they are "vehicles” for potential virulence factors with intracellular targets in the host, which parasitic wasps use to counter host immunity.

### Role of Transported Factors

The role and intracellular targets of proteins transported in lamellocytes (and maybe plasmatocytes whose function may be altered although no apparent morphological changes were observed) remain largely hypothetical. LbGAP interacts *in vitro* and in yeast two-hybrid experiments with Rac1 and Rac2 (27), two Rho GTPases essential for the encapsulation process (34, 50, 51), but we still await validation of these targets in lamellocytes. LbGAP2, as well as other members of the venom LbGAP family, bears mutations in the active site as well as sites important for the interaction with GTPases (25). The association of these others RhoGAPs with venosomes still needs to be tested. The way in which LbGAP2 (and maybe the other RhoGAPs) affects the physiology of lamellocytes and whether they have evolved new functions related to their mutations is still enigmatic. The high concentration of LbGAP and LbGAP2 in the venosomes could in any case facilitate their targeted quantitative delivery and their action at the cellular level. There is still a lack of information on whether venosomes may be found in other host tissues than lamellocytes and plasmatocytes. Induced apoptosis of hematopoietic precursors in the lymph gland of *Drosophila* by *L. heterotoma* and *L. victoriae* envenomation has been suggested (22), but this has recently been reexamined (52). Improving our approach using fluorescently labeled venosomes combined with larval clarification could help solving this question in the future.

### Species Specific Venosome Entry

We finally evaluated the level of venosomes entry in lamellocytes from various species of *Drosophila* to assess the specificity and thus the conservation of the involved mechanism(s). The observation of a much higher entry level of ISm LbGAP (and thus venosomes) in the lamellocytes of *D. melanogaster* and *D. simulans* [two species on which the parasitoid succeeds (43)], compared with *D. yakuba* and *D. suzukii* [very low or no parasitism success, (43–45, 53)] suggests the existence of a specific or, at least, more effective mechanism of targeting/uptake in the lamellocytes of these two species. Elucidating such mechanism will include identifying the mode of entry of the venosomes and the potential lamellocytes membrane receptors involved. Our data then suggest that a new “vesicle-cell” interaction level may take part in the host-parasitoid specificity in addition to behavioral aspects, physiological adequacy and venom factors/targets interaction.

In conclusion, extracellular vesicles are most likely vectors used by many pathogens or parasites to propagate or transport virulence factors. We have clearly shown here that some parasitoid wasps use very unusual vesicles, assembled extracellularly in their venom apparatus, to transport and target specific potential virulence factors important for successful parasitism. Thus, these wasp species represent an interesting model to explore the role of EVs in inter-species communication, in particular to control the immune response of the host, a field that has not been investigated yet.

## Data Availability

All datasets generated for this study are included in the manuscript/Supplementary Files.

## Author Contributions

BW performed light and fluorescence microscopy, venosomes purifications and labeling, microinjection, SDS-PAGE, and Western blotting. MR and A-NV performed transmission electron microscopy. OP set up the larval clarification protocol and helped with confocal microscopy. EG and SL performed venosomes purification and encapsulation assays. BW, J-LG, and MP wrote the manuscript. J-LG and MP designed and coordinated the work. All authors have read and approved the final manuscript.

### Conflict of Interest Statement

The authors declare that the research was conducted in the absence of any commercial or financial relationships that could be construed as a potential conflict of interest.
