# Supplementary material for: Venom Atypical Extracellular Vesicles as Interspecies Vehicles of Virulence Factors Involved in Host Specificity: The Case of a Drosophila Parasitoid Wasp
Source: Front Immunol. 2019 Jul 17;10:1688. doi: 10.3389/fimmu.2019.01688 (PMC6653201; doi:10.3389/fimmu.2019.01688)
Supplement: Supplementary file 1 [file Data_Sheet_1.pdf]

S1 figure: Histological overview of the venom apparatus

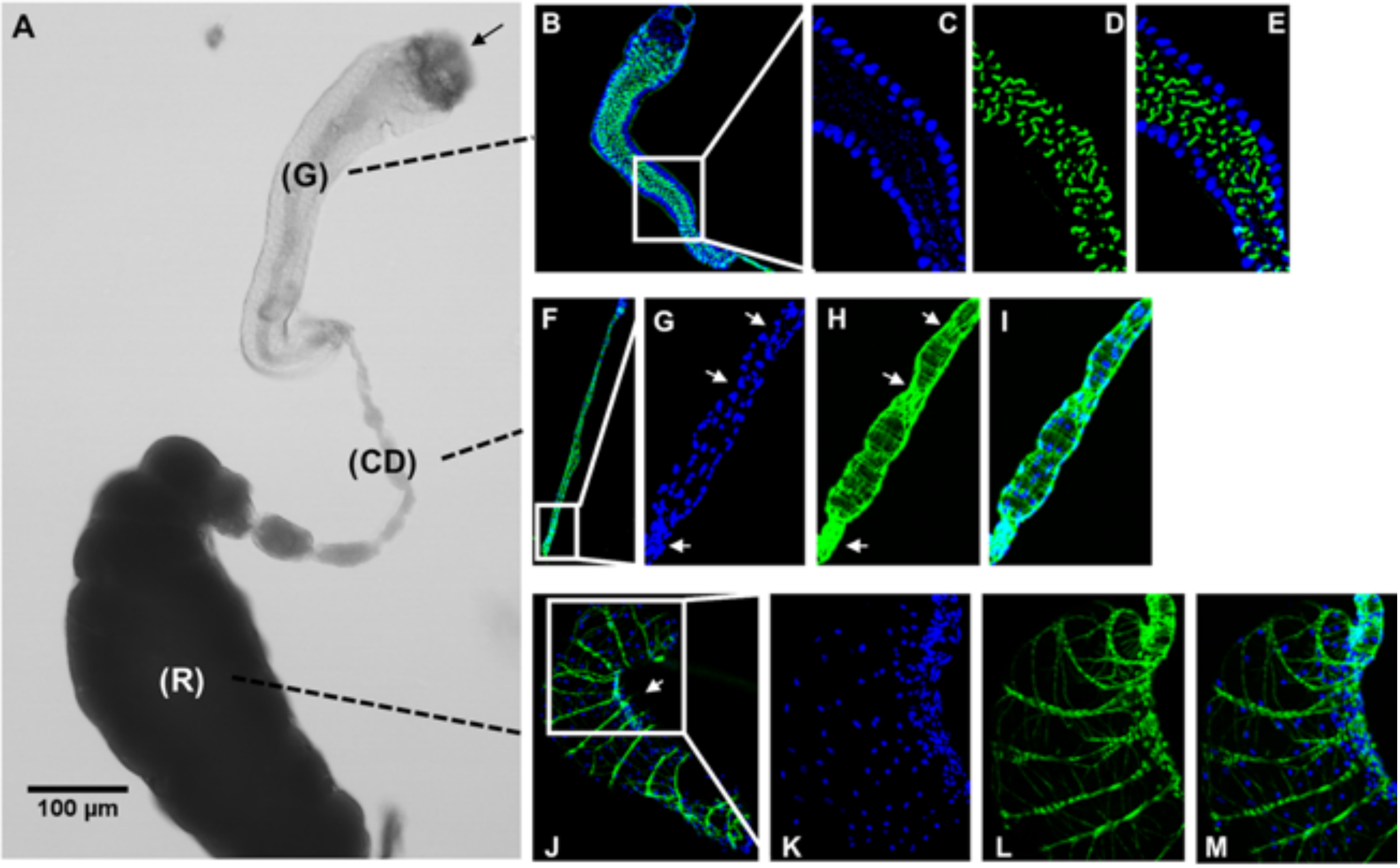

Panel A: The *L. boulardi* venom apparatus consists in an elongated venom gland (also named long gland, (panel A, (G)), linked by a connecting duct (panel A, (CD)) to a large reservoir (panel A, (R)). The distal end of the gland is enlarged and terminated by a structure called “nose”, where a darkish material can be observed (Panel A, arrow). From this end, begins the collecting canal of the central gland which decreases rapidly in width, and then remains of constant diameter until the junction with the connecting duct. Panel B-E (C, D, E enlargement of B): based on cell nucleus morphology observed by confocal microscopy (DAPI-labeling), two types of cells are present in the gland (in C): a first type, with a large nucleus, located at the periphery, which represents glandular secretory cells, and a second type with a small nucleus that forms the “intimal layer” that surrounds the central collecting canal. Green phalloidin strongly labeled the actin-rich microvilli lining the upper part of the secretory canal found in each secretory cell (in D). The lower part of this cell canal has no microvilli and is less reactive since it is covered by a cuticle-like material formed by the extension from the cells of the intimal layer, that also coats the wall of the connecting duct [17]. In E, merged picture from B and C showing the cell nucleus and the cell canal position.

Less is known about the *Figitid* connecting duct (CD) and reservoir (R). The CD is about 100 to 150 µm long and varies in diameter (panel A and F-I (G, H, I enlargement of F)). All cell nuclei seem to have the same size, but zones with restricted diameter may contain a higher number of cells (G, arrows) and show a denser actin network (H, arrows). The actin pattern suggests the presence of a circular muscular layer all along the duct, which may allow circulation of the fluid to the reservoir by peristaltic contractions. The muscular layer is still present at the reservoir junction and continues on the reservoir (J-M, (K, L, M enlargement of J). The reservoir is a very large, non-uniform structure (700-800 µm long with a diameter of 150-200 µm) made of a series of enlargements separated by restricted regions (visible in A). On the side of the reservoir where the CD make junction (in J, indicated by an arrow), we observed a higher density of cells and muscle fibers based on actin staining (in L). The density of the cell nuclei was very low in its lateral and opposite sides, with only one layer of cell nucleus, each well separated from the others (in K). Each nucleus seems at the center of a large flat cell forming a continuous monolayer at the surface of each enlargement (as judged also by the confocal microscopy observation and TEM cross section through the reservoir (not shown)). Each enlargement is itself separated from its neighbors by what appears as a chord of muscular cells (L-M). These muscular chords could play several roles, such as facilitating and controlling venom ejection at oviposition, or maintaining a homogeneous composition by mixing newly formed and older venom. They could also girdle the reservoir to limit its volume in the abdominal cavity of the wasp.
