# Supplementary material for: Venom Atypical Extracellular Vesicles as Interspecies Vehicles of Virulence Factors Involved in Host Specificity: The Case of a Drosophila Parasitoid Wasp
Source: Front Immunol. 2019 Jul 17;10:1688. doi: 10.3389/fimmu.2019.01688 (PMC6653201; doi:10.3389/fimmu.2019.01688)
Supplement: Supplementary file 2 [file Data_Sheet_2.PDF]

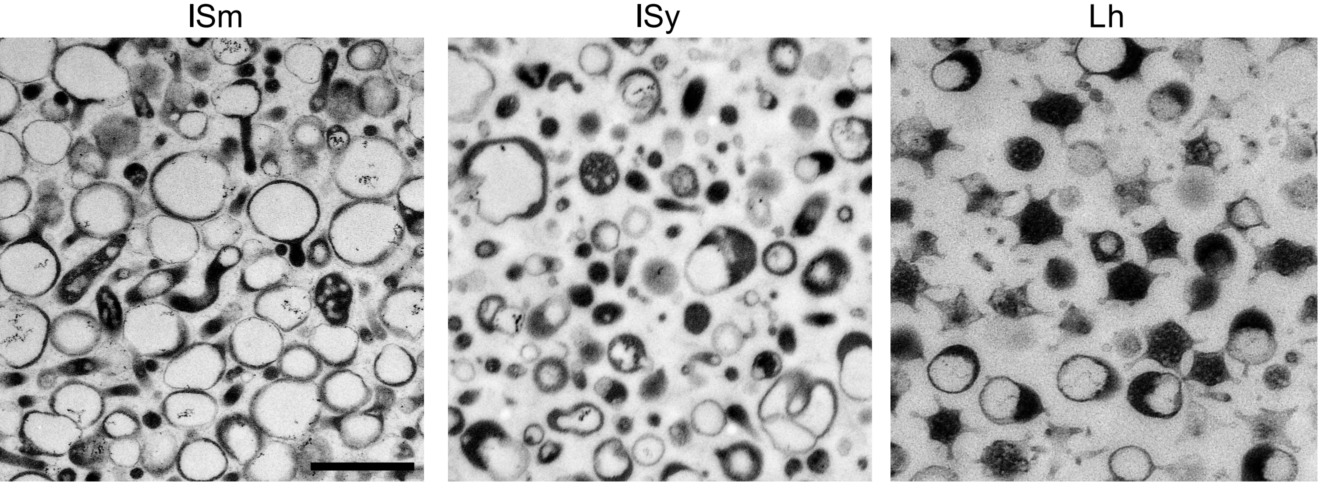


**S2 Figure. Electron microscopy of *Leptopilina* 15,000g venosomes pellet cross-section.** Venom of *L. boulardi* ISm and ISy strains and *L. heterotoma* (Lh) were centrifuged at 15,000 g and the pellets were treated for electron microscopy as described in mat. et met. Many *L. heterotoma* venosomes show a stellate shape due to extending spikes while none of the ISm and ISy venosomes have a similar shape. In the three preparations empty venosomes with one side or the membrane enlarged are visible. Bar: 1µm.
