# Supplementary material for: Venom Atypical Extracellular Vesicles as Interspecies Vehicles of Virulence Factors Involved in Host Specificity: The Case of a Drosophila Parasitoid Wasp
Source: Front Immunol. 2019 Jul 17;10:1688. doi: 10.3389/fimmu.2019.01688 (PMC6653201; doi:10.3389/fimmu.2019.01688)
Supplement: Supplementary file 3 [file Data_Sheet_3.PDF]

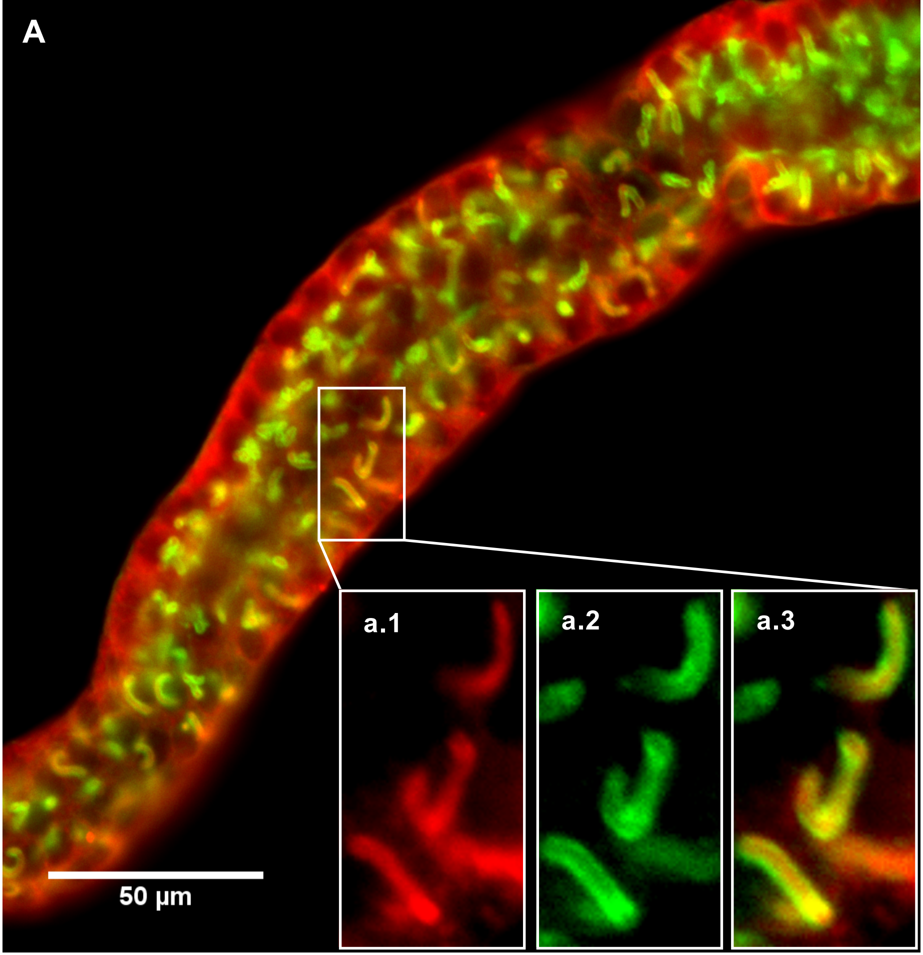

S3 Figure: Immunolocalization by confocal microcopy of LbGAP2 *in L. boulardi* ISm venom gland. Panel A shows the merged picture obtained from the gland labeled in red for LbGAP2 and counterstained for actin with green phalloidin. Panels a1, a2 and a3 show an enlarged region corresponding to the microvilli region of the secreting canal from the gland cell: LbGAP (a1, red) and actin (a2, green) show a strong coimmunolocalization on the merged picture (a3, yellow).
