# Supplementary material for: Venom Atypical Extracellular Vesicles as Interspecies Vehicles of Virulence Factors Involved in Host Specificity: The Case of a Drosophila Parasitoid Wasp
Source: Front Immunol. 2019 Jul 17;10:1688. doi: 10.3389/fimmu.2019.01688 (PMC6653201; doi:10.3389/fimmu.2019.01688)
Supplement: Supplementary file 4 [file Data_Sheet_4.PDF]

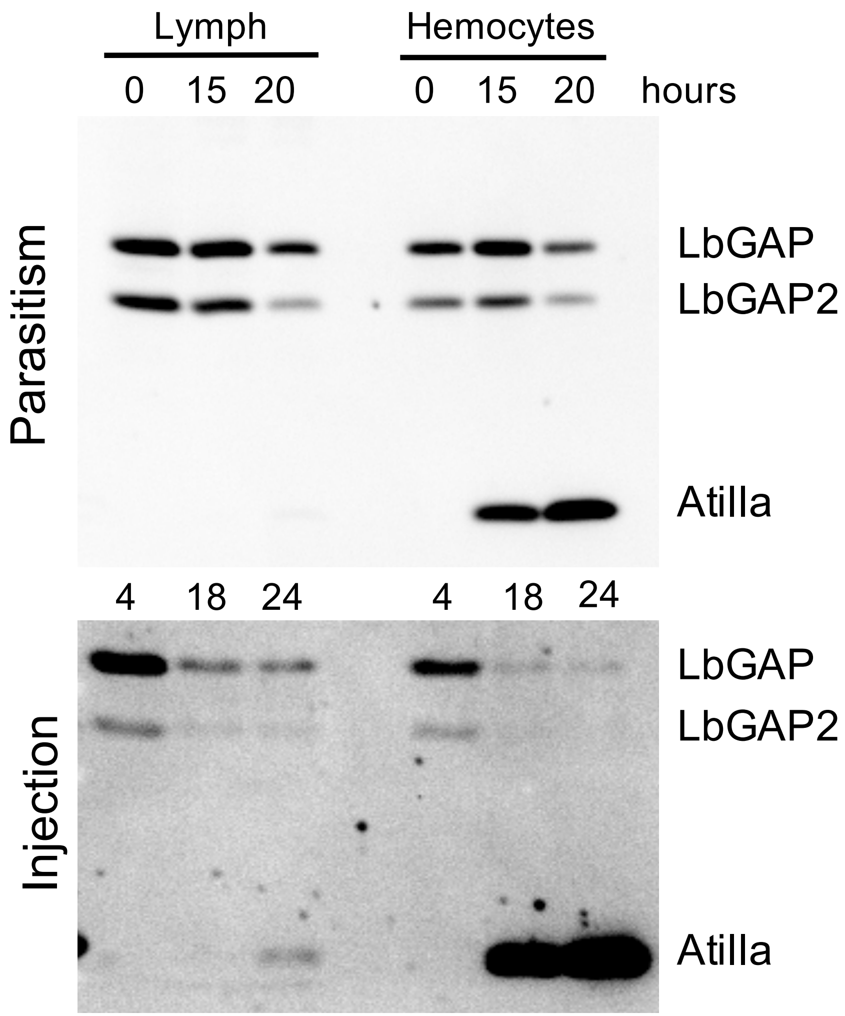

S4 Figure: Persistence of virulence factors in hemolymph and hemocytes. Upper panel: *D. melanogaster* YR larvae hemolymph was collected at 0, 15 and 20h after the end of parasitism assay by *L. boulardi* ISm female and the hemocytes separated by centrifugation. The cell free hemolymph (Lymph) and washed hemocytes pellets (hemocytes) were separated on 12% SDS-PAGE, transferred to nitrocellulose and probed with the anti-LbGAP, anti-LbGAP2 and anti-Atilla (a lamellocyte marker) antibodies. At all times, LbGAP and LbGAP2 were present in clear hemolymph and cells pellets while lamellocytes increased strongly in number after 15h, as shown by the Atilla reaction. Lower panel: the same experiment was done 4, 18 and 24h after purified venosomes injection. Although the amount of circulating and cellular LbGAP and LbGAP2 were lower at 18 and 24h, they were still present. Increase in Atilla at 18h in cells pellets indicated that injection initiated also the increase in lamellocytes number.
