# Supplementary material for: Venom Atypical Extracellular Vesicles as Interspecies Vehicles of Virulence Factors Involved in Host Specificity: The Case of a Drosophila Parasitoid Wasp
Source: Front Immunol. 2019 Jul 17;10:1688. doi: 10.3389/fimmu.2019.01688 (PMC6653201; doi:10.3389/fimmu.2019.01688)
Supplement: Supplementary file 5 [file Data_Sheet_5.PDF]

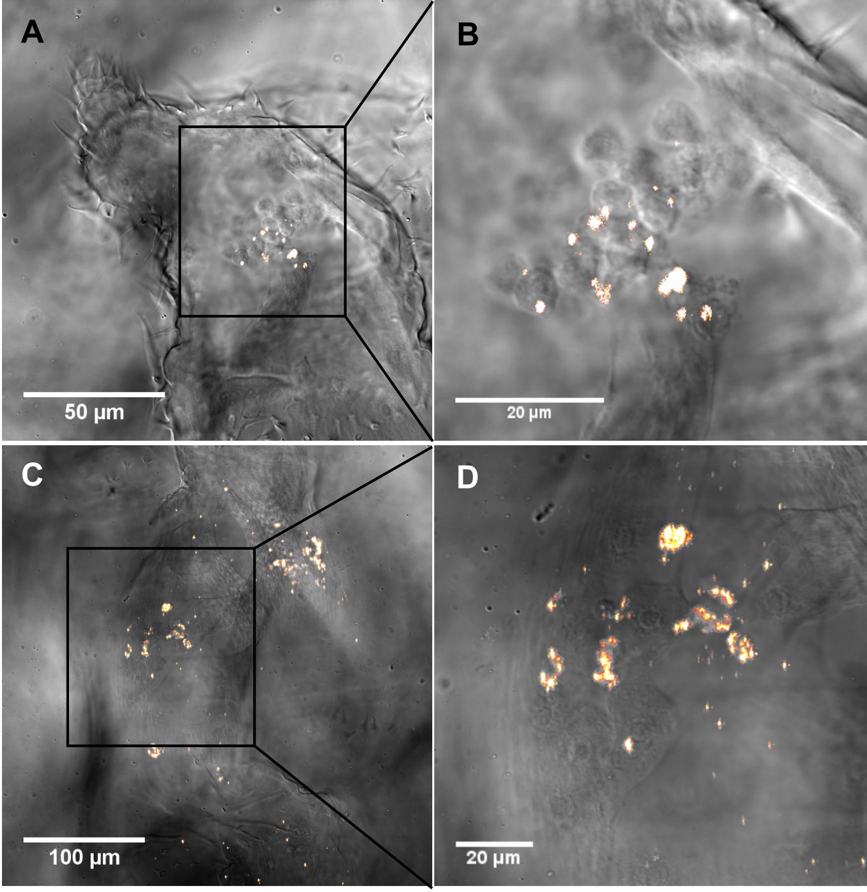

S5 Figure: Venosomes immunolocalization in *Drosophila* larvae sessile hemocytes after tissue clearing. *D. melanogaster* YR larvae were fixed 18h after injection of fluorescently labeled venosomes and treated to clarify their tissues. Confocal microscopy shows fluorescence spots at the subcuticular level in the head (A) and on the back of the larvae (B). When the inserts in A and B were enlarged (C, D), the fluorescently labeled venosomes were found in small round cells of about 10 µm (C) and in large flat cells of 30-40 µm (D) respectively, representing certainly sessile plasmatocytes and lamellocytes.
