# Supplementary material for: Venom Atypical Extracellular Vesicles as Interspecies Vehicles of Virulence Factors Involved in Host Specificity: The Case of a Drosophila Parasitoid Wasp
Source: Front Immunol. 2019 Jul 17;10:1688. doi: 10.3389/fimmu.2019.01688 (PMC6653201; doi:10.3389/fimmu.2019.01688)
Supplement: Supplementary file 6 [file Data_Sheet_6.pdf]

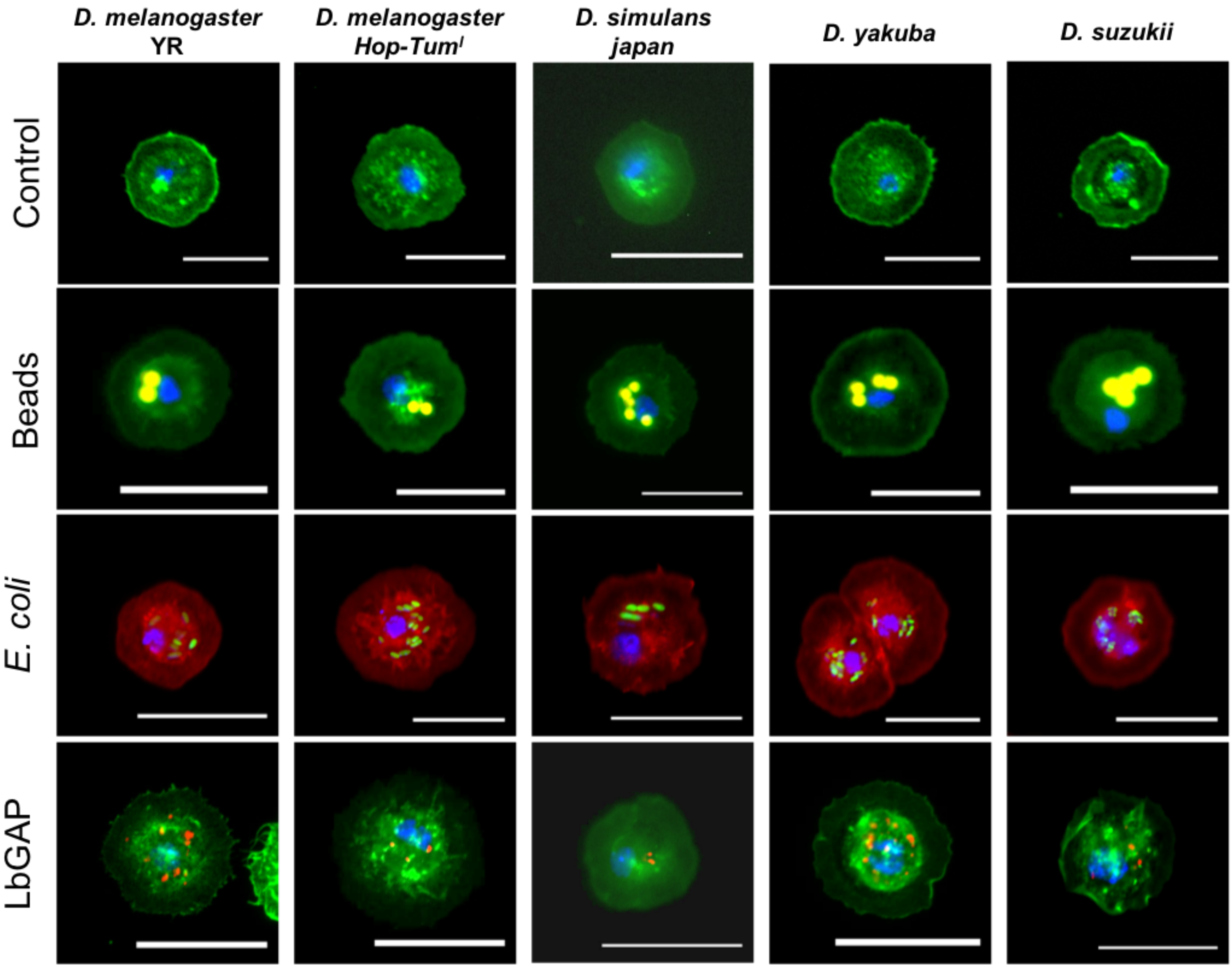

S6 Figure: Phagocytic properties of plasmatocytes from different Drosophila species. First row: plasmatocytes observed in hemolymph of 18h parasitized *D. melanogaster* YR and hop^{Tum-l}, *D. simulans* Japan, *D. yakuba* and *D. suzukii*. Plasmatocytes were observed 4 hours after injection of 18h parasitized L2 larvae with fluorescent latex beads (Second row), green fluorescent *E. coli* (third row) and venosomes (fourth row) to evaluate their phagocytic properties. Actin labelled with green (rows 1, 2 and 4) or red (row 3) fluorescent phalloidin; in blue, nucleus stained with DAPI. Row 4, phagocytosed LbGAP immunolocalized in red. Bar 20µm.
